# Custom CGH array profiling of copy number variations (CNVs) on chromosome 6p21.32 (HLA locus) in patients with venous malformations associated with multiple sclerosis

**DOI:** 10.1186/1471-2350-11-64

**Published:** 2010-04-28

**Authors:** Alessandra Ferlini, Matteo Bovolenta, Marcella Neri, Francesca Gualandi, Alessandra Balboni, Anton Yuryev, Fabrizio Salvi, Donato Gemmati, Alberto Liboni, Paolo Zamboni

**Affiliations:** 1 Section of Medical Genetics, Department of Experimental and Diagnostic Medicine, University of Ferrara, Ferrara, Italy; 2 Ariadne Genomics Inc., Rockville, USA; 3 Department of Neurology, Bellaria Hospital, Bologna, Italy; 4 Interdepartmental Vascular Disease Center, University of Ferrara, Ferrara, Italy

## Abstract

**Background:**

Multiple sclerosis (MS) is a complex disorder thought to result from an interaction between environmental and genetic predisposing factors which have not yet been characterised, although it is known to be associated with the HLA region on 6p21.32. Recently, a picture of chronic cerebrospinal venous insufficiency (CCSVI), consequent to stenosing venous malformation of the main extra-cranial outflow routes (VM), has been described in patients affected with MS, introducing an additional phenotype with possible pathogenic significance.

**Methods:**

In order to explore the presence of copy number variations (CNVs) within the HLA locus, a custom CGH array was designed to cover 7 Mb of the HLA locus region (6,899,999 bp; chr6:29,900,001-36,800,000). Genomic DNA of the 15 patients with CCSVI/VM and MS was hybridised in duplicate.

**Results:**

In total, 322 CNVs, of which 225 were extragenic and 97 intragenic, were identified in 15 patients. 234 known polymorphic CNVs were detected, the majority of these being situated in non-coding or extragenic regions. The overall number of CNVs (both extra- and intragenic) showed a robust and significant correlation with the number of stenosing VMs (Spearman: r = 0.6590, p = 0.0104; linear regression analysis r = 0.6577, p = 0.0106).

The region we analysed contains 211 known genes. By using pathway analysis focused on angiogenesis and venous development, MS, and immunity, we tentatively highlight several genes as possible susceptibility factor candidates involved in this peculiar phenotype.

**Conclusions:**

The CNVs contained in the HLA locus region in patients with the novel phenotype of CCSVI/VM and MS were mapped in detail, demonstrating a significant correlation between the number of known CNVs found in the HLA region and the number of CCSVI-VMs identified in patients. Pathway analysis revealed common routes of interaction of several of the genes involved in angiogenesis and immunity contained within this region. Despite the small sample size in this pilot study, it does suggest that the number of multiple polymorphic CNVs in the HLA locus deserves further study, owing to their possible involvement in susceptibility to this novel MS/VM plus phenotype, and perhaps even other types of the disease.

## Background

Although multiple sclerosis is the most prevalent neurological disease in the young adult population, it is catalogued as a neurodegenerative disorder of unknown aetiology [1]. Indeed, despite the proposal of inflammatory, infective, and autoimmune factors as pathogenic agents in this disease, their links with its aetiology still remain to be elucidated [2,3]. Nonetheless, genetic studies on twins and siblings suggest that susceptibility genes may play a role in predisposition. Indeed, candidate gene and whole genome association studies, as well as CNV detection on SNP-based arrays involving more than hundred thousand markers, have identified several possible susceptibility loci in the human genome. The HLA locus on 6p21.32 is the most confidently associated of these [4-9], among a few others of uncertain statistical significance [10,11]. However, even when controls are accurately randomised, undetectable errors may occur, especially linked to the geographical origins of the population and known differences in SNPs density, depending on the various human chromosomes or even genomic regions involved. These errors may inflate the apparently significant differences between patients and controls (genomic inflation) generating false positives or negatives and impeding true recognition of the associated loci [11].

As recently reported [11], and as recommend by the Wellcome Trust Case Control Consortium [12,13], in order to circumvent this issue, allowing unbiased data to be collected and replication of the associations in the identified loci to be performed, an enormous number of individuals have to be analysed and functional studies are required.

All the studies into the genetics of MS performed so far have been carried out on SNP-based arrays. However, although SNPs do contribute to inter-individual variation across the genome, it is now well recognised that copy number variations (CNVs), typically ranging from 1 kb to several Mb, also influence genetic variations and disease susceptibility [14,8]. It is evident that CNVs account for more nucleotide variations between individuals, and, furthermore, the functional significance of these variations might be more immediate, especially if they are located within genes, regulatory regions or known imprinted regions, since the possible consequence of genome imbalance(s) may be more easily interpretable.

In fact, the development of robust high throughput platforms based on comparative genomic hybridisation (CGH) capable of identifying thousands of genomic variations has greatly improved research in this direction, as recently demonstrated in the case of Amyotrophic Lateral Sclerosis, a major neurodegenerative disorder in which non-polymorphic sub-microscopic duplications and deletions seem to be frequent in sporadic cases [15].

Recently, we described the peculiar association of chronic cerebrospinal venous insufficiency (CCSVI) in patients with MS [16]. CCSVI is due to stenosing venous malformations (VM) which affect the azygous and the jugular veins, leading to significant anomalies in cerebral venous outflow haemodynamics [16-20]. Insufficient cerebral venous drainage represents a mechanism potentially related to increased iron stores, suggesting a pathogenic role in the progression or even pathogenesis of this disease [21-23].

Taking into account these premises, the aim of our pilot study was to use an innovative CGH array to investigate the occurrence of CNVs underlying genome imbalances in the major locus (HLA, chromosome 6p21) associated with MS. In order to sub-select a specific phenotype, we decided to recruit patients with the "plus" phenotype (VM-CCSVI and MS). The objective was thus to use this patient category to explore the genomic configuration of the locus in question, thereby seeking to identify specific regions for further investigation in a wider population.

## Methods

### Subjects

Fifteen patients affected by relapsing-remitting MS, diagnosed according to the revised McDonald criteria [24], were recruited for the study, and their Expanded Disability Status Scale (EDSS) [25] and Multiple Sclerosis Severity Scores (MS-SS) [26,27] were determined. In our population, MS was associated with CCSVI venous malformation, documented by a sequential Colour-Doppler/selective venography protocol [16]. In fact, Colour Doppler allowed us to determine the number of anomalous parameters linked to CCSVI, as well as the Venous Haemodynamic Insufficiency Severity Score (VHISS) based on the number of venous segments exhibiting reflux or blocked flow [18,28]. The clinical and demographic characteristics of the selected patients are given in the Additional file 1 Table S1. Informed consent was obtained from all patients in the study.

DNA from the 15 patients was extracted by a protocol recommended by Agilent. Highly concentrated DNA was checked for quality using a NanoDrop (260/280 ratio = 1.8 and 260/230 ratio = 2.0), and DNA integrity was evaluated on agarose gel at 1% in TBE 1×.

### Ethical aspects

All the experimental research that is reported in the manuscript was performed with the approval of the University of Ferrara Ethical Committee (Document n. 7, approved by the 27^th ^of May 2004). The research carried out on humans was in compliance with the Helsinki Declaration http://www.wma.net/en/30publications/10policies/b3/index.html.

### Statistical Analysis

Clinical data are given as median and interquartile range. For genotype-phenotype correlations, patients with an overall number of CNVs within the 90^th ^percentile were considered. Genotype-phenotype correlations were further analysed by means of both the Spearman rank correlation test and linear regression analysis, including evaluation of the slope and X and Y intercept, followed by a Run Test. P values of < 0.05 were considered to be significant.

### HLA locus typing

HLA-DRB1 low resolution SSP typing was performed using commercial kits (Biotest DRB SSP Kit, Lot B812151) in all 15 patients studied.

### Microarray design, hybridization and data analysis

MS-CGH microarray design was carried out using the web-based Agilent eArray database, version 5.4 (Agilent Technologies, Santa Clara, CA) [29], as previously performed [30]. The high density aCGH search function in eArray was used to turn the genomic region chr6:29,900,001-36,800,000 (March 2006, human reference sequence, NCBI Build 36.1, hg18) into a probe set by selecting the maximum number of exonic, intronic and intragenic 60 mer oligonucleotide CGH probes available in the database. This set included 43102 probes that were used to reach the array format of 4 × 44 K, creating four identical 44 K arrays on a single slide for simultaneous analysis of four different samples.

This platform, termed MS-CGH, is a high-density microarray with a resolution of one probe per 160 bp which permits rapid determination of the molecular profile, identifying the presence of heterozygous or homozygous copy number variations (CNVs) in the genomic region studied.

The platform informations have been submitted to the online data repository Gene Expression Omnibus (GEO) [31], with accession number GPL10049.

Labelling and hybridisation were performed following the protocols provided by Agilent (Agilent Oligonucleotide Array-Based CGH for Genomic DNA Analysis protocol v5.0), as described elsewhere [30]. The array was analysed with the Agilent scanner and Feature Extraction software (v9.1). A graphical overview was obtained and data analysis performed using DNA Analytics software (v4.0.36). The standard set-up of the ADM-2 statistical analysis in this software package was employed for identification of duplications and deletions. In this set-up, and in the case of autosomal genes, heterozygous deletions are visualised with values of minus 1, and homozygous deletions as minus infinite (-4 in CGH analytics). The corresponding values for heterozygous and homozygous duplications are plus 0.5 and plus 1, respectively. At least 4 consecutive non-overlapping probes reaching these values were required for a positive reading, together with an absence of known SNPs in the region covered by the relevant probes. All 15 patients were assessed in duplicate in order to confirm the validity of the results.

### Bioinformatics analysis of gene networks

Pathway analysis and literature mining was performed using Pathway Studio software from Ariadne Genomics Inc. The Pathway Studio database contains millions of regulatory and interaction events from all PubMed abstracts, and more than 350,000 full-text articles extracted by MedScan natural language processing technology. We employed the "Build Pathway" navigation tool in Pathway Studio to locate literary evidence supporting the functional association of measured genes with angiogenesis and other processes linked to blood vessel formation, as well as to immunity and neurodegeneration.

## Results

### CGH-ARRAY data

**Figure 1:**
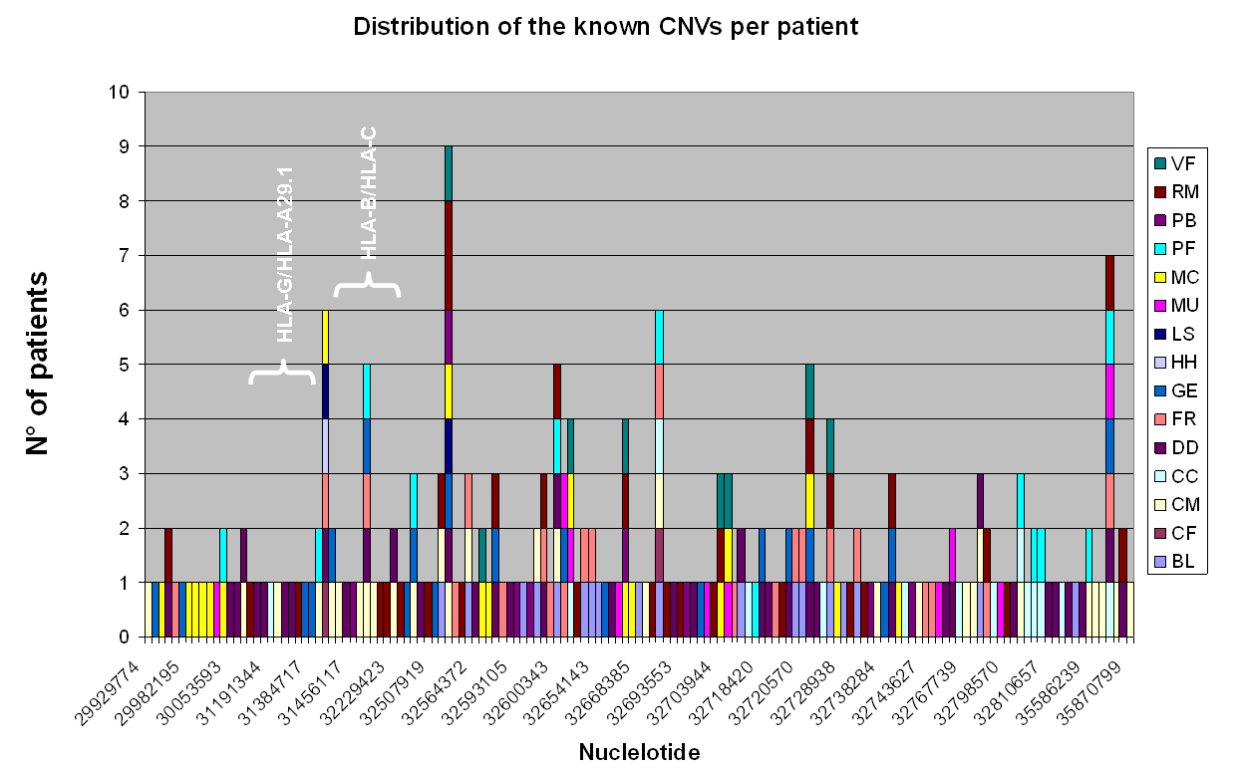
**Genomic distribution of the known CNVs along the HLA locus among the patients studied**. In the graph the starting nucleotide for each CNV is reported. The HLA gene regions are highlighted in white.

Comparison with the CNV database revealed 234 known polymorphic CNVs in the 15 patients [32], thereby confirmed that the HLA locus is highly polymorphic in terms of genomic imbalance, as expected considering its known high density of SNPs. The distribution of the CNVs among patients, both in terms of number and density, is shown in Figure 1.

Additional file 2 Table S2 reports all the CNVs identified in patients. An example CGH profile, corresponding to patient PF, is shown in Additional file 3 Figure S1.

The complete CGH datasets have been submitted to the online data repository Gene Expression Omnibus [31], under accession number GSE20334.

### Genotype-phenotype correlation: statistical analysis

#### CNV profiling

**Figure 2:**
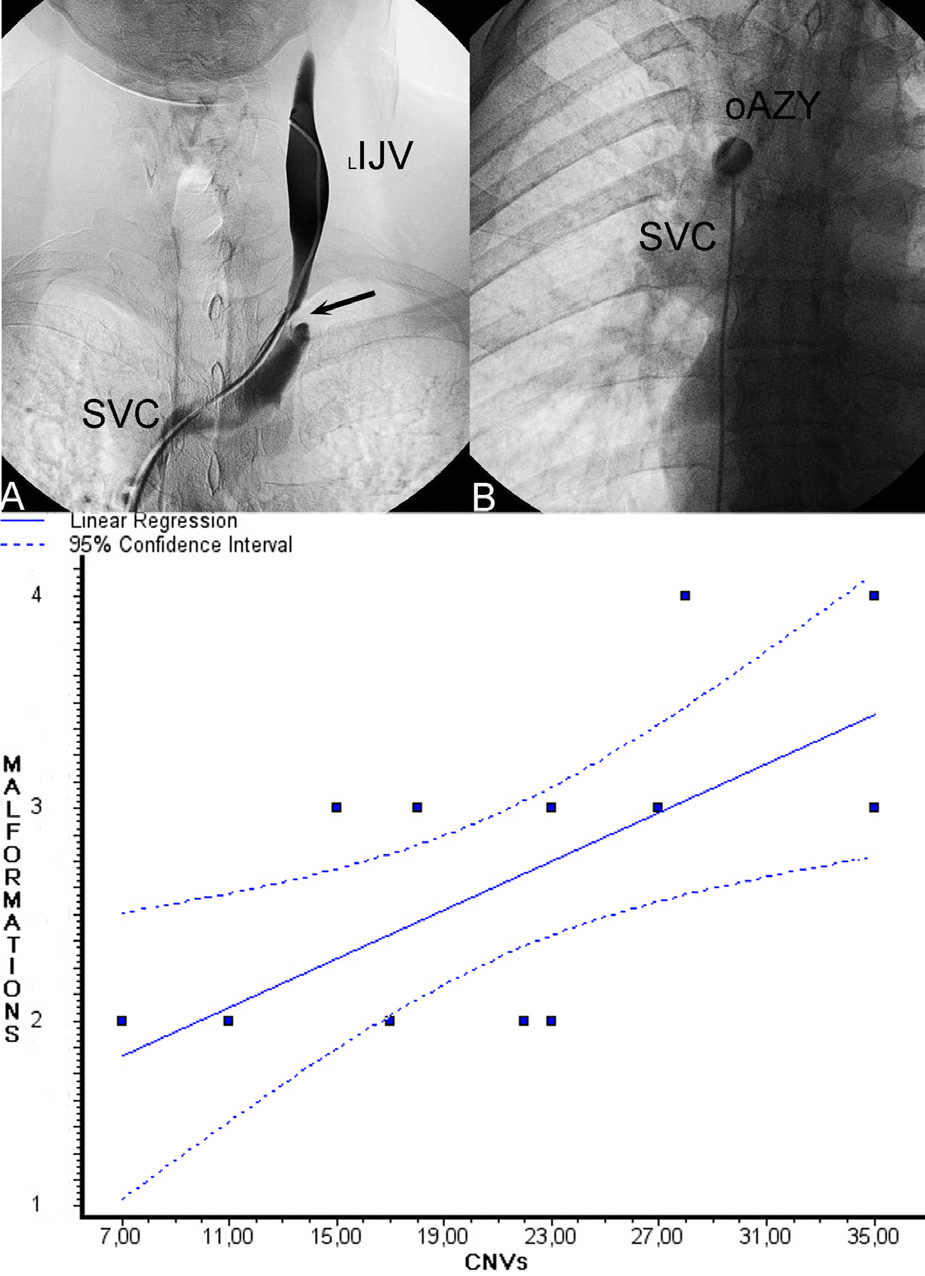
**Stenosing venous malformation associated to MS and genotype-phenotype correlation**. Top Panel: Exemplification of stenosing venous malformation associated to MS. Significant stenosis (arrow) of the left internal jugular vein (L IJV). B) Membranous obstruction of the outlet of the azygous vein (o AZY) into the superior vena cava (SVC). Bottom Panel) Linear regression analysis. A significant correlation between the number of CNVs and the number of venous malformations detected by means of selective venography was found (r = 0.6577, p = 0.0106).

**Figure 3:**
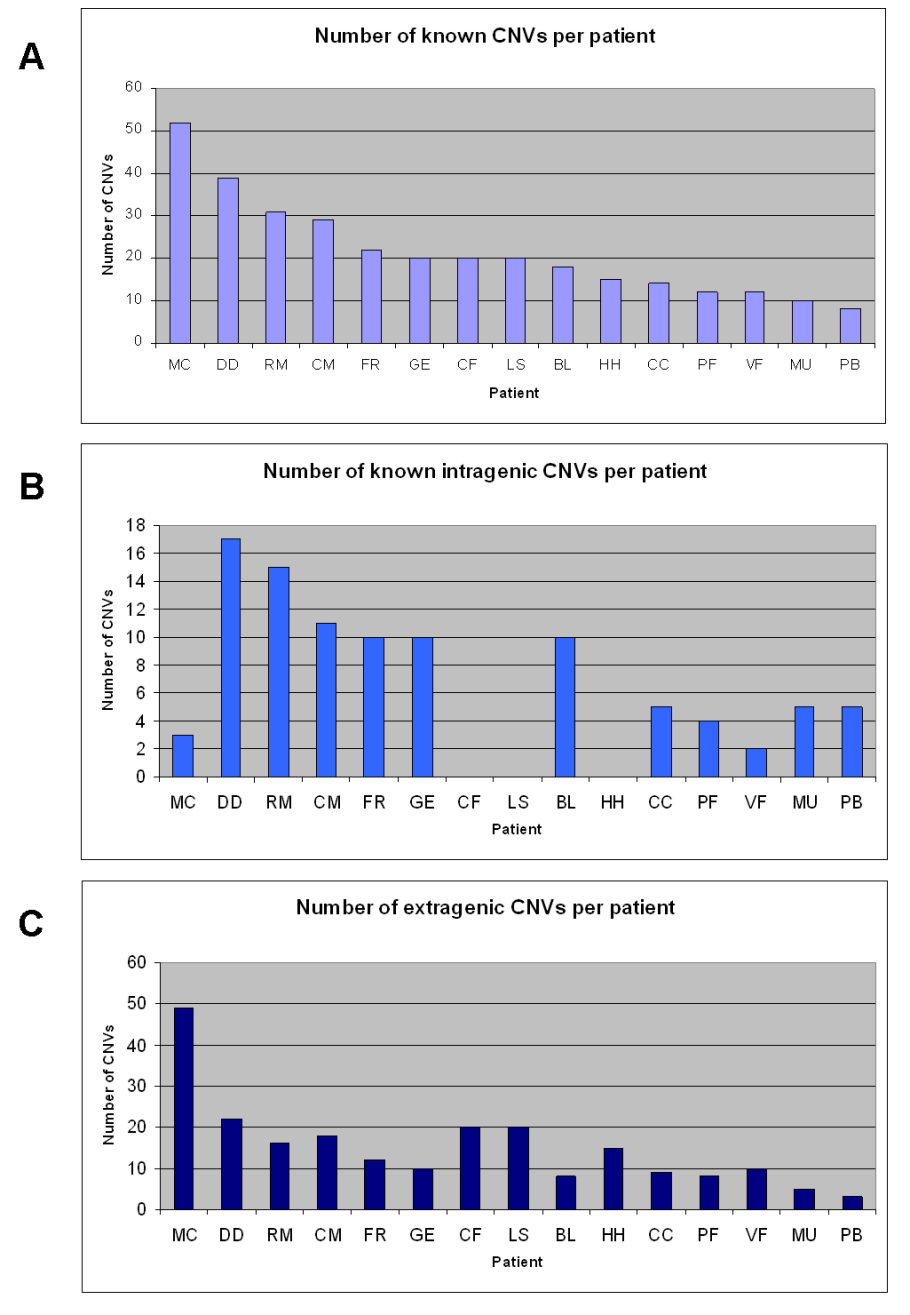
**Number of total (A), intragenic (B) and extragenic (C) known CNVs reported in the Database of Genomic Variants (http://projects.tcag.ca/variation) per patient**.

The number of CNVs within the 90^th ^percentile was correlated with the clinical parameters of the patients (Figure 2, top panel, Additional file 1 Table S1). Fourteen patients out of the those coded as MC (52 CNVs vs. median (IR) 23 (13) of our patient population) fell within the 90^th ^percentile, and were therefore further analysed by both the Spearman rank correlation test and linear regression analysis (Additional file 1 Table S1). Both analyses demonstrated a significant and firm correlation between the number of CNVs and the number of stenosing venous malformations identified (Spearman: r = 0.6590, p = 0.0104; linear regression analysis r = 0.6577, p = 0.0106) (Figure 2 bottom panel and Figure 3).

No correlation was found between extragenic or intragenic CNVs and the number of stenosing VMs, respectively. No additional correlations were found between CNVs and the number of anomalous Doppler haemodynamic parameters, or with the VHISS. No correlation was found between patient phenotypes, either for MS and VM, and the presence of specific CNVs, CNV haplotypes or CNV distribution.

#### HLA typing

Statistical analysis did not yield significant results. The HLA-DRB1*15 class region, known to be a locus with a major contribution to the risk of MS [33], did not strongly occur in our population, and seemed to be unrelated to VM clinical signs or CNV number (see Additional file 1 Table S1).

Since the patients' parents were not available for study, haplotypes and consequently allele phases could not be established.

### Pathways and gene network functional bioinformatics analysis

211 genes were contained within the region covered by our CGH array.

**Figure 4:**
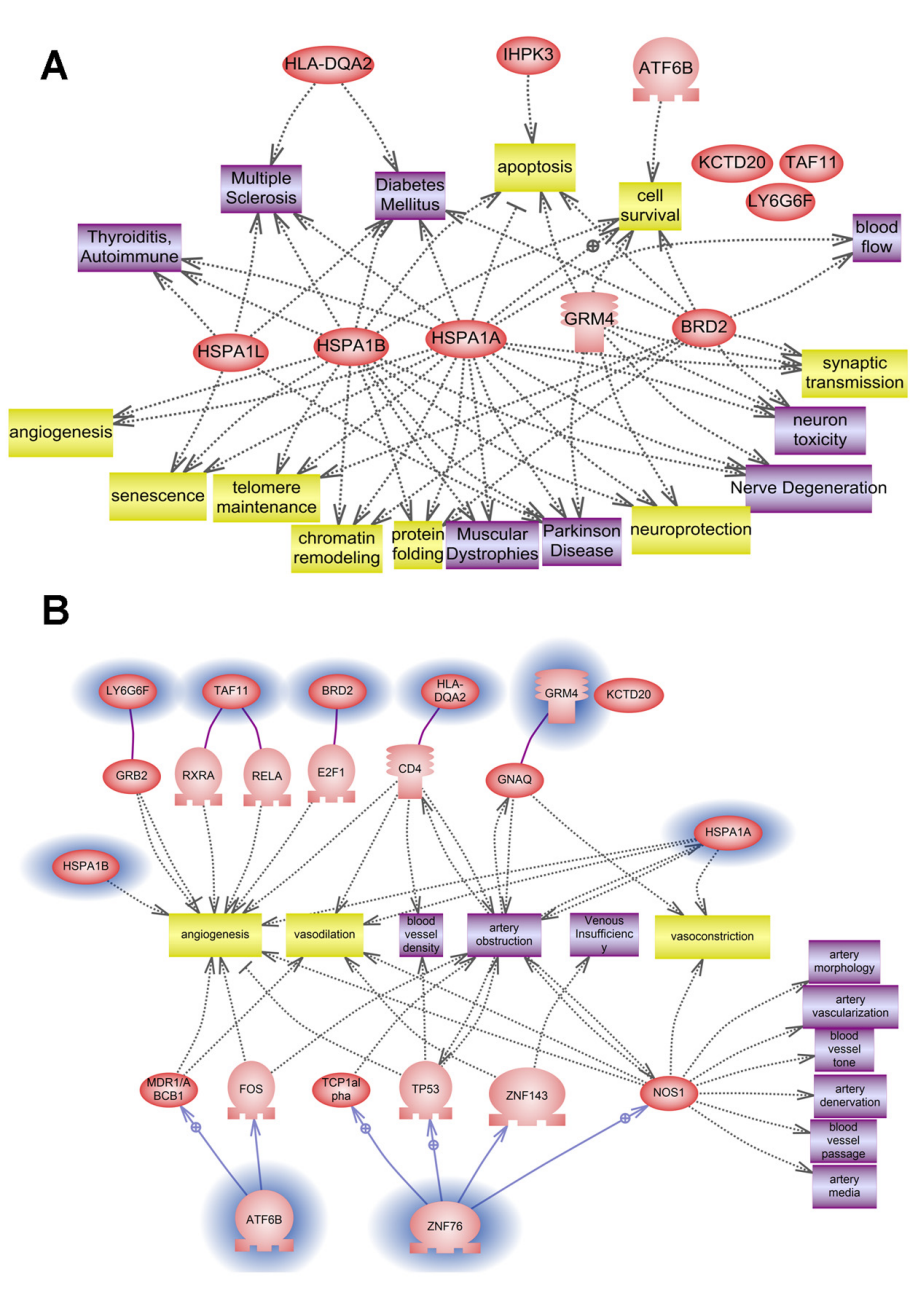
**Functional links of the known genes within the HLA locus networking in neurodegeneration, multiple sclerosis and immunity disorders (A) and angiogenesis and venous formation pathways (B)**.

Since the phenotype in question is characterised by multiple sclerosis and venous malformation, we applied a bioinformatics tool to select genes known to be involved in angiogenesis and venous development, as well as those linked to multiple sclerosis, immunity and neurodegeneration. Interestingly, several genes have been linked to these processes. HSPA1L and HSPA1A have been correlated with MS, diabetes and other immunity disorders, as well as regulatory functions such as chromatin remodelling, neuroprotection, protein folding, and regenerative-degenerative tools like neurodegeneration, neuron toxicity, cell survival, synaptic transmission and even aging factors (senescence and telomere maintenance). The gene GRM4 also seems to interact with many proteins linked to MS (Figure 4A)

By focusing on specific functional pathways, for example angiogenesis, and considering the plus phenotype of our patients (venous malformation), we obtained a more selective puzzle of interactions (Figure 4B). GRB2 and HSPA1A and B genes directly act on angiogenesis, TAF11 is known to be involved in artery passage, and the E2F1 transcription factor is known to be an angiogenesis positive inducer in hepatitis and cancer. Interestingly, HLA-DQA2 may also be implicated in angiogenesis through its interaction with CD4.

## Discussion

We recently described the CCSVI/VM phenotype associated with MS, which might participate in iron accumulation in the brain, a feature known to be present in several neurodegenerative diseases, including MS [34-36].

We designed a locus-specific CGH array in order to explore the occurrence of CNVs in the HLA region in 15 patients with the peculiar association of CCSVI/VM and MS phenotype.

CNVs are very abundant in the human genome, being involved in genetic variation among populations in a similar fashion to SNPs. Innovatively, this strategy was adopted instead of SNP-based arrays, as the latter tend to suffer from false positive and false negative results, in addition to the fact that no algorithm able to unequivocally detect genomic variants of less than about 30 Kb seems to be currently available.

However, CGH CNV detection also has inherent limitations, such as probe density, and failure of hybridisation due to the presence of SNPs in the probed region. In addition, it is widely recommended that each novel CNV detected by CGH be validated by an alternative method (for example RealTime PCR or qPCR). Nevertheless, being aware of the limit of confidence of CNV identification by CGH, the advantage of searching for CNVs by this technique was the possibility of directly correlating known, validated CNVs with a potential function, which can be related to imbalance either of specific gene(s) or of genomic regulatory regions. In fact, the CGH approach allows fine mapping of the identified CNVs in non-genic regions, which may be correlated to gene regulatory functions, epigenetic changes or other non-coding functions. Indeed, the potential impact on the expression regulation of many genes by genomic "perturbation" of one (or more) specific genomic region(s) is intriguing, as it tentatively implicates it in a variety of different complex phenotypes. This is the reason why this genetic model applies very well to polygenic or multi-factorial diseases [37].

As expected, we detected a high number (234) of known CNVs, thereby confirming that the HLA region is very rich in structural variations, in addition to its known high polymorphism in terms of SNPs.

Analysing the distribution of the polymorphic CNVs identified in patients, we observed a peak of CNV numbers within the HLA region. Outside this specific region however, the number of CNVs per patients remained high, though with variable distribution.

We also genotyped the HLA-DRB1 region in our patients. Statistical analysis failed to show any correlation between the presence of the HLA-DRB1*15 allele and VM-related clinical signs. It is well known that SNPs or complex polymorphism density is haplotype-dependent within the human Major Histocompatibility Complex (MHC) [38].

However, in order to link the CNV profile and the HLA haplotype, phasing both polymorphism types is mandatory. In addition, no studies have yet fully characterised either CNVs (array-based) or SNPs in the whole genomic region of the HLA locus. For this reason, although the frequency of HLA-DRB1*15 in our patients' cohort seems to be lower than the one reported in classic MS, it is not possible to conclude whether the VM phenotype has a distinct association with the HLA haplotype, especially when one considers our small patient number. This seems to be a major goal for future studies.

Interestingly, while no statistically significant association was found between CNV type or distribution and patient phenotype, the overall number of CNVs showed a significant correlation with the number of stenosing malformations demonstrated by venography in the extracranial segments of the cerebrospinal veins. However, the major shortcoming of this pilot study is the dimension of the sample, which should be expanded in the future to strengthen the as yet unconfirmed significance of our findings. The small number of patients also affected further sub-analysis, and both the extragenic and the intragenic component of the CNVs were not found to be associated to the phenotype VM. Moreover, no correlations were discovered between CNVs and either the number of anomalous Doppler haemodynamic parameters or the VHISS.

Nonetheless, the phenotype studied here, correlated with the CNVs, is strongly associated to MS (OR 43, p < 0.0001) [16]; we speculate that the presence of VM may contribute to the increase in iron accumulation in MS as a pathogenic component of the disease [36]. The hampered cerebral venous return consequent to extracranial venous malformations is peculiar to MS, and was not found in a miscellany of patients affected by other neurodegenerative disorders characterized by iron stores, such as Parkinson's, Alzheimer's, and amyotrophic lateral sclerosis [16,18,36]. Theoretically, venous haemodynamic overload may facilitate local microbleeding following damage to vein or venule walls, becoming a distinctive mechanism of iron deposition in MS, as we recently demonstrated [36].

Taken as a whole, our data, though preliminary, suggest that the number of polymorphic CNVs in the HLA region did correlate with the number of VMs in our patient cohort. Since HLA is the only region consistently associated with the disease, further studies are certainly needed to discriminate whether the CNV findings are specific for MS patients with venous malformations or, instead, are similarly observed in the general MS population, regardless of the presence of vascular malformations.

In addition, the region studied contains 211 known genes. Using a functional bioinformatics tool, we identified many genes interacting in both neurodegenerative and angiogenesis circuits. Notably, HSPA1L, HSP1A and HSP1B and the HLA-DQ2 gene network in both pathways. Heat-shock proteins (HSPs) represent a group of regulatory proteins involved in a variety of processes, including immunity and angiogenesis [39,40]. In particular HSPA1L expression is modulated by ETS1 transcription factor and by SP100, a nuclear autoimmune antigen. Interestingly, genes negatively regulated by ETS1 and up-regulated by SP100, such as HSPA1L, have anti-migratory or anti-angiogenic properties [41].

MS possesses a recognised major heritable component, since its susceptibility is associated with the MHC class II region, especially the HLA-DRB5*0101-HLA-DRB1*1501-HLA-DQA1*0102-HLA-DQB1*0602 haplotypes, which dominate genetic contribution to MS susceptibility [42]. Interestingly, HLA-DQA2 is known to be involved in pro-inflammatory CD4(+) T-cell-mediated autoimmune diseases such as MS and type 1 diabetes [43]. CD4 is also a very well known inhibitor of tumour angiogenesis [44], thus supporting a link between the two pathways. The interpretation of the pathway interaction is obviously complex, but it does suggest biological and functional links among these genes as well as, intriguingly, between angiogenesis and immunity.

## Conclusions

In conclusion, we present an exploration of CNVs, identified by CGH-based methodology, in a small group of patients with associated MS and VM. We identified 234 known CNVs, and determined that the distribution of CNVs along the HLA region showed no peculiar topography in the patients studied.

We found that the overall number of CNVs correlates significantly with the venous malformative phenotype. Consistently with the general significance of CNVs, putatively involved in regulation of gene expression, this finding is interesting, since it may lend weight to the possibility that the number of structural variations within regulatory regions represents a genomic "perturbation" which increases susceptibility for the VM phenotype associated with MS we describe. Obviously, confirmation of these findings will require further studies aimed at comparing CNV profiles in patients with the MS/VM phenotype to those in patients presenting MS alone. This will distinguish peculiar association(s), and potentially disclose common/different underlying genotypes. Regarding specific candidate genes whose expression could potentially be disturbed by genomic imbalance or "perturbation", pathways analysis suggested that genes involved in angiogenesis and immunity could be proteins worthy of further investigation.

Moreover, we hope that our custom array will be useful for other studies, perhaps to identify structural changes in the numerous disorders proven to be linked to the HLA locus, including MS.

## Abbreviations

CNVs: Copy number variations; CGH: Comparative genomic hybridization; SNPs: Single nucleotide polymorphisms; VM: Venous malformations; EDSS: Expanded disability status scale; MS-SS: Multiple sclerosis severity score; CCSVI: Chronic cerebrospinal venous insufficiency; VHISS: Venous haemodynamic insufficiency severity score; VH: Venous haemodynamic criteria.

## Competing interests

The authors declare that they have no competing interests.

## Authors' contributions

MB designed and validated the custom CGH-array; MN contributed to the patients' genomic analysis; AY performed the networking genes pathway analysis via Ariadne software; FS studied the patients from neurological aspects; AB performed HLA genotyping, DG collected DNA samples, AL and PZ planned, performed and interpreted the vascular studies; FG scientifically revised the manuscript; AF designed the study and wrote the manuscript, AF and PZ revised the manuscript and approved the final version. All the authors read and approved the final manuscript.

## Pre-publication history

The pre-publication history for this paper can be accessed here:

http://www.biomedcentral.com/1471-2350/11/64/prepub

## Supplementary Material

Additional file 1 **Patients Population Demographics, Clinical Parameters, HLA DRB1 haplotype and CNVs number**. Top table: EDSS: Expanded Disability Status Scale (the most widely-used disability score). MS-SS: Multiple Sclerosis Severity Score (score expressing the tendency of MS to progress over the years). VH: Venous Haemodynamic Criteria (number of anomalous haemodynamic parameters detected by colour Doppler protocol), VHISS: venous haemodynamic insufficiency severity score [34]. VM: venous malformations (number of stenosing malformations affecting the cerebrospinal veins, detected by selective venography). CNVs: copy number variations. Bottom table: Summary of clinical characteristics, HLA DRB1*5 typing and number of CNVs of each patient studied. Click here for file

Additional file 2 **Genomic distribution of CNVs for each patient**. Genomic coordinates of the known CNVs identified for each patient. Column F shows the genes where any CNVs are localized and column H the identification number of known CNVs as described in the database of Genomic Variants. Click here for file

Additional file 3 **CGH profile of the HLA locus**. Example of a CGH profile in patient PF, showing duplications (red dots) and deletions (green dots)- Light blue bars in the lower part of the figure represent genes, while the known polymorphic CNVs are reported with their identification number (Database of Genomic Variants at http://projects.tcag.ca/variation/). Below is shown the map in Mbases of the locus with the distribution and density of CNVs. Click here for file
