# Supplementary material for: Custom CGH array profiling of copy number variations (CNVs) on chromosome 6p21.32 (HLA locus) in patients with venous malformations associated with multiple sclerosis
Source: BMC Med Genet. 2010 Apr 28;11:64. doi: 10.1186/1471-2350-11-64 (PMC2880319; doi:10.1186/1471-2350-11-64)
Supplement: Additional file 1 — Patients Population Demographics, Clinical Parameters, HLA DRB1 haplotype and CNVs number. Top table: EDSS: Expanded Disability Status Scale (the most widely-used disability score). MS-SS: Multiple Sclerosis Severity Score (score expressing the tendency of MS to progress over the years). VH: Venous Haemodynamic Criteria (number of anomalous haemodynamic parameters detected by colour Doppler protocol), VHISS: venous haemodynamic insufficiency severity score [34]. VM: venous malformations (number of stenosing malformations affecting the cerebrospinal veins, detected by selective venography). CNVs: copy number variations. Bottom table: Summary of clinical characteristics, HLA DRB1*5 typing and number of CNVs of each patient studied. [file 1471-2350-11-64-S1.DOC]

|  | **Multiple sclerosis relapsing remitting**  **n°15**  **Median**  **(IR)** |
| --- | --- |
| **Age, years** | **36**  **(12)** |
| **Sex M/F** | **7/8** |
| **Disease duration, years** | **6**  **(9)** |
| **EDSS** | **1.5**  **(1)** |
| **MS-SS** | **2.6**  **(3.8)** |
| **Number of Fulfilled VH Criteria** | **3**  **(1)** |
| **VHISS** | **9**  **(2)** |
| Number of VM | **2**  **(1)** |
| **Total CNVs** | **23**  **(13)** |

| **PATIENT CODE** | **SEX** | AGE | **NUMBER OF VM** | **MS**  **DISEASE DURATION YEARS** | **EDSS** | **MS-SS** | **Number of Fulfilled VH Criteria** | **VHISS** | **HLA DRB1** | **Number of CNVs** |
| --- | --- | --- | --- | --- | --- | --- | --- | --- | --- | --- |
| **PB** | **F** | **36** | **2** | **15** | **7** | **8,17** | **3** | **6** | **DRB1*01, *11** | **8** |
| **RM** | M | **31** | **3** | **3** | **1,5** | **3,34** | **2** | **6** | **DRB1*11** | **31** |
| **DD** | **M** | **46** | **4** | **3** | **2** | **4,82** | **3** | **9** | **DRB1*07, *11** | **39** |
| **GE** | **M** | **46** | **2** | **6** | **1** | **1,13** | **4** | **9** | **DRB1*04, *11** | **20** |
| **BL** | **M** | **40** | **3** | **14** | **1,5** | **1,03** | **4** | **9** | **DRB1*07** | **18** |
| **CC** | **M** | **32** | **3** | **4** | **1** | **1,45** | **5** | **8** | **DRB1*14, *15** | **14** |
| **FR** | **M** | **46** | **3** | **12** | **1** | **0,64** | **3** | **10** | **DRB1*07** | **22** |
| **PF** | **F** | **27** | **2** | **4** | **1** | **1,45** | **2** | **7** | **DRB1*04, *13** | **12** |
| **CM** | **F** | **46** | **2** | **15** | **4** | **5,09** | **3** | **10** | **DRB1*04, *10** | **23** |
| **MU** | **M** | **40** | **2** | **9** | **1** | **0,88** | **3** | **9** | **DRB1*08, *15** | **10** |
| **CF** | **F** | **30** | **2** | **5** | **1,5** | **2,6** | **2** | **8** | **DRB1*10, *13** | **29** |
| **HH** | **F** | **36** | **3** | **3** | **1,5** | **3,34** | **4** | **12** | **DRB1*07, *15** | **15** |
| **LS** | **F** | **25** | **4** | **3** | **2** | **4,82** | **3** | **8** | **DRB1*01, *11** | **20** |
| **VF** | **F** | **36** | **2** | **6** | **0,5** | **0,25** | **3** | **5** | **DRB1*03** | **12** |
| **MC** | **F** | **37** | **2** | **3** | **1,5** | **3,34** | **4** | **9** | **DRB1*03** | **52** |

**Supplementary Table S1. Patients Population Demographics, Clinical Parameters, HLA DRB1 haplotype and CNVs number.**
