# Supplementary figures and images for: Custom CGH array profiling of copy number variations (CNVs) on chromosome 6p21.32 (HLA locus) in patients with venous malformations associated with multiple sclerosis
Source: BMC Med Genet. 2010 Apr 28;11:64. doi: 10.1186/1471-2350-11-64 (PMC2880319; doi:10.1186/1471-2350-11-64)

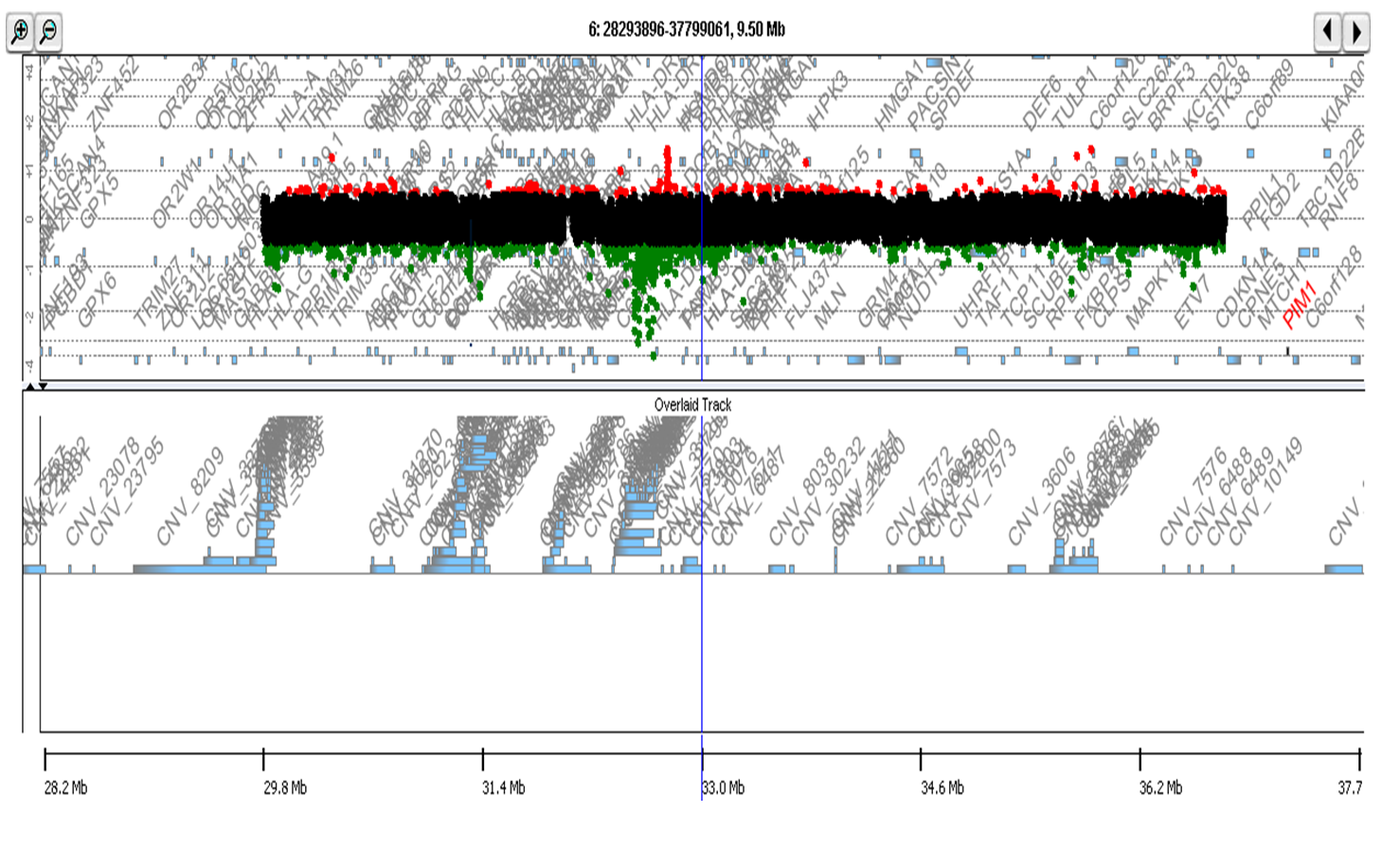

Supplement: Additional file 3 — CGH profile of the HLA locus. Example of a CGH profile in patient PF, showing duplications (red dots) and deletions (green dots)- Light blue bars in the lower part of the figure represent genes, while the known polymorphic CNVs are reported with their identification number (Database of Genomic Variants at http://projects.tcag.ca/variation/). Below is shown the map in Mbases of the locus with the distribution and density of CNVs. [file 1471-2350-11-64-S3.PNG]
